# Management of cancer treatment-related fatigue in advanced breast cancer patients: an expert committee’s opinion

**DOI:** 10.3389/fonc.2025.1617600

**Published:** 2025-10-09

**Authors:** Stéphanie Bécourt, Sophie Abadie-Lacourtoisie, Thierry Calvat, Isabelle Coudurier-Curveur, Frédéric Fiteni, Benoîte Mery, Guillaume Meynard, Florian Scotté, Pauline Vaflard, Laurent Zelek

**Affiliations:** ^1^ Department of Medical Oncology, Centre Oscar Lambret, Lille, France; ^2^ Department of Medical Oncology, Institut cancérologie de l'ouest site Paul Papin, Angers, France; ^3^ Association Juris Santé, Lyon, France; ^4^ Collectif 1310/Patients en réseau, Paris, France; ^5^ Association Les Lyonnes de Tatooine, Brindas, France; ^6^ Medical Oncology Department, University Hospital of Nîmes, Nîmes, France; ^7^ Département of Medical Oncology, Centre Léon-Bérard, Lyon, France; ^8^ Centre hospitalier universitaire de Besançon, Oncologie, Besançon, France; ^9^ Gustave Roussy Cancer Campus, Villejuif, France; ^10^ Department of Medical Oncology, Institut Curie, Paris, France; ^11^ Medical and Thoracic Oncology Department, Hopital Avicenne, Assistance Publique – Hôpitaux de Paris, Bobigny, France

**Keywords:** cancer-related fatigue, cancer treatment-related fatigue, advanced breast cancer, metastatic breast cancer, physical activity, adapted physical activity, patient education

## Abstract

Cancer-related fatigue (CRF) is a frequent and complex adverse event associated with advanced breast cancer (ABC). CRF intensity and impact on a patient’s daily life are often exacerbated by cancer treatments. This specific manifestation, known as cancer treatment related fatigue (CTRF), begins with treatment onset and can persist beyond its course. CTRF is not unavoidable; its effect can be reduced through careful management and differential diagnosis from CRF. This article aims to review current recommendations for assessing and managing fatigue and present an expert opinion on priority actionable actions for evaluating and alleviating fatigue in ABC patients. In addition, to better understand the current standard of care and management options for ABC patients with fatigue, a quantitative survey was conducted from July to September 2023 through online standardized questionnaire containing identical questions between oncologists (N = 43) and ABC patients (N = 132) in France. Results confirm fatigue’s complexity and multidimensional nature. Insufficient time and lack of communication during consultations contribute to ineffective diagnosis and management of fatigue, highlighting the need for improvement through better communication.

## Introduction

Cancer related fatigue (CRF), also referred to as asthenia (1), is a common and frequently disabling symptom among patients diagnosed with advanced breast cancer (ABC). Cancer treatment related fatigue (CTRF) a specific manifestation of CRF, typically occurs at the initiation of anticancer treatment protocols and is characterized by its persistence after rest. CTRF is frequently associated with chemotherapy, immunotherapy or endocrine therapy (2) and can persist for months or even years following treatment completion (3, 4). While the terms fatigue, asthenia, weakness, exhaustion, malaise, and tiredness are frequently used interchangeably by both physicians and patients, it is important to note that only fatigue and asthenia are officially defined as medical conditions in the Medical Subject Heading (MeSH) Index (5). CRF often appears before treatment starts and may persist after the cessation of the treatment (5). At present, the reported prevalence of CRF in advanced cancer patients ranges from 50% to 71%. However, precise estimates are challenging due to variations in the populations studied and the associated methodological approaches (6). CRF often occurs in tandem with other troublesome symptoms such as pain, sleep disturbance and depression (5). As a result of the multifaceted nature of CRF symptoms, multiple definitions exist within the literature. It is commonly agreed among authors that CRF encompasses a distressing, persistent, subjective sense of physical, emotional and/or cognitive tiredness or exhaustion related to cancer or its treatment that is not proportional to recent physical activity (PA) and interferes with usual functioning (7–9). Moreover, CRF assessment involves a subjective experience that requires the patient to express themselves directly through self-reporting (7).

Innovative therapies for ABC, such as the new generation of antibody-drug conjugates (ADCs), have significantly improved survival rates in terms of both progression-free survival and overall survival. Consequently, patients undergoing these treatments may experience extended periods of CTRF, necessitating dedicated long-term care plans, the focus of this paper (3). CTRF frequently remains unnoticed and untreated, in part because of its multifactorial origins. The clinical expression of CTRF varies depending on tumor stage and treatment (7, 8, 10, 11). Growing evidence indicates that a gradual decline in PA and subsequent deconditioning before the start of new anticancer treatments are likely to play a significant role in triggering chronic fatigue symptoms (7, 11, 12). These symptoms may be further exacerbated by concurrent factors such as disease progression and cancer-related adverse events and also by external causes such as impact on family or social life (11–13). Deconditioned states and CTRF have also been linked with psychological distress, which in turn undermines quality of life (QoL) and potentially reduces overall survival (9). In addition to improving fatigue, physical exercise has been shown to reduce side effects during and after treatment. The PREFERABLE-EFFET study demonstrated an improvement in fatigue and health-related quality of life (HRQOL) in metastatic breast cancer patients after 9 months of supervised exercise (14).

To address the high prevalence and severity of CTRF and the associated distress suffered by ABC patients, a group of French breast cancer experts came together to provide an overview of fatigue screening and management in ABC patients in Europe. This article aims to outline current guidelines for assessing and managing CTRF while presenting the Working Group’s opinions on priority actionable actions for evaluating and alleviating fatigue in ABC patients to enhance their QoL.

## Current state of CRF and CTRF management in Europe

Despite significant progress in understanding CRF and its treatment-related counterpart, a specific diagnostic algorithm has not been definitively established (7). The most recent Clinical Practice Guidelines from the European Society for Medical Oncology (ESMO) advocate for the screening of CTRF through patient self-reporting, using a 10-point numerical rating scale (NRS) or a visual analog scale (VAS) to gauge fatigue (7). However, these methods have inherent limitations, primarily stemming from their incapacity to encompass the myriad of symptoms that often accompany or impact fatigue. Another challenge inherent in CTRF detection pertains to the comparison of fatigue assessments over time, regardless of the screening tool used (7). Although several instruments for measuring fatigue have been developed, their predominant use remains confined to clinical research (15). Diagnostic assessment in European guidelines, including French recommendations for cancer patient supportive care, emphasizes the importance of identifying contributing and comorbid conditions amenable to treatment strategies (7, 11). This necessitates a comprehensive evaluation encompassing the patient’s fatigue history, a thorough medical examination, an evaluation of the underlying malignant disease, a systemic review, a mental status examination, and a defined set of laboratory tests. All modifiable contributing factors should be managed and periodically reassessed through ongoing fatigue screening, which should be consistently integrated into the continuum of care (7, 11).

To address the symptoms and underlying factors of CTRF, a comprehensive approach involving both non-pharmacological interventions (such as adapted PA, nutritional management, and strategies targeting cognitive, behavioral, educational, motivational aspects) and pharmacological treatments (including optimization of anticancer drug doses and management of associated toxicities) might be considered (7, 11, 16). Accumulating evidence in the context of advanced cancer suggests that PA is an effective therapeutic modality for improving fatigue, physical and psychosocial functions, quality of life, body composition, and sleep quality (17). Regular PA is also associated with lower cancer incidence and mortality, as well as a lower rate of tumor recurrence (18). As evidenced by a prospective study involving 103 patients with metastatic or locally recurrent breast cancer (mean age: 53.8 years; mean follow-up: 60.43 months), engaging in one or more hours of moderate PA per day was associated with a 23% reduction in mortality compared to individuals who participated in less than one hour of daily exercise (19). Recommendations for aerobic PA include engaging in at least 150 minutes per week of moderate activities (e.g. 30 minutes of brisk walking a day; 3 times a week “aerobic” exercise; 30 minutes, 2 times a week strength exercises) or at least 75 minutes per week of vigorous activities (such as brisk or very brisk walking; 25 minutes, 2 times a week aerobic exercise; 25 minutes, 1 time a week strength exercises), or a combination thereof. The mechanisms underlying the antitumor effects of exercise remain incompletely understood. These effects might be associated with exercise’s direct impact on tumor cells, which could involve the inhibition of tumor cell proliferation, induction of apoptosis, upregulation of tumor suppressor genes, and the exertion of anti-inflammatory effects (8, 20). PA may also play a role in mitigating cytokine dysregulation induced by CRF/CTRF and reducing the levels of tumor necrosis factor (18). In addition, exercise has the capacity to enhance immune function, thereby contributing to its potential antitumor activity (8, 20). Accordingly, moderate-intensity physical exercise punctuated by rest intervals, along with aerobic and functional resistance training, is recommended for non-cachexic ABC patients with CRF or CTRF (7, 11, 16, 21). However, the presence of CRF/CTRF appears to function as a physiological deterrent to PA engagement among patients. Furthermore, a lack of social support, suboptimal guidance from healthcare professionals (HCPs), and limited access to fitness facilities equipped with knowledgeable staff for patient education represent substantial obstacles to patient involvement in PA (22, 23).

Psychosocial interventions, including psychosocial counseling, psychotherapy, and mind-body interventions, assist patients in assessing their CRF and adapting to their prevailing condition and treatment (7, 11). To empower patients, contemporary guidelines incorporate educational programs aimed at equipping them with the knowledge and skills necessary to understand the multifactorial nature of CTRF, its influencing factors, and its patterns during and post-cancer treatment (7, 11, 24). As illustrated in a meta-analysis of 14 studies involving 2,213 participants with various cancer types, educational strategies appear to exert a limited effect on fatigue intensity but have a moderate impact on reducing fatigue-related distress (24). This suggests that counseling programs should be implemented alongside other interventions (7, 25). It is noteworthy that patients often express a need for educational interventions and guidance from medical staff. A recent study involving 2,508 German cancer patients revealed that approximately 60% of respondents lacked information concerning fatigue, underscoring the need for further research to enhance the delivery of patient education and its seamless integration into routine care (26).

## Methods

### Composition and scope of the working group

The Working Group chaired by Professor L. Zelek, was involved from the beginning of the project, in the formulation of the program schedule and selection of appropriate individual and organizational participants. Participants were selected on the basis of their demonstrated expertise in the subject matter addressed by the program, as well as their ability to represent a wide spectrum of healthcare interests in the diagnosis and treatment of ABC and associated fatigue. Upon the confirmation of their participation, individual interviews were conducted to obtain insights into their clinical practices related to the management of CTRF. Subsequently, participants convened to approve the final program schedule and to create the framework of the quantitative survey mirroring the experiences of both oncologists and metastatic breast cancer patients in the management of CTRF which would be used in this study after validation by all members of the scientific committee and the patient associations cited below.

### Quantitative analysis of fatigue management in France

To better understand the current standard of care provided for ABC patients with CRF/CTRF and the management options available to them, an online survey in French was implemented. Study participants were recruited via the associations Collectif 13/10, Europe Donna France, Les Lionnes de Tatooine and AFSOS from a database of around 2,000 patients. Between July and September 2023, metastatic breast cancer patients undergoing treatment and oncologists caring for patients with MBC, replied using a self-completed questionnaire (**Supplementary Data 1, 2**). This comprised a series of open or closed questions, 47 for patients and 34 for doctors, which allowed data reflecting both patients’ and physicians’ perspectives of fatigue and its management in metastatic breast cancer to be collected. After a brief screening section, both groups responded to questions related to their experience of the care pathway (9 questions for patients and 7 for oncologists), the management of fatigue (19 and 17 questions, respectively) and difficulties encountered by oncologists (2 questions for each group). In each of these sections there were a number of answers which allowed mirror-image analysis (**Supplementary Appendix 3**). For some questions, a numerical scale from 0 (not at all) to 10 (always) was employed, while others proposed single or multiple-choice answers. Only significant and relevant differences (95%; +/- 3pts) were indicated. A selection of the results most relevant to the aims of this paper, namely to reach a consensus on priority actionable recommendations for evaluating and alleviating fatigue in ABC patients, thus improving their treatment response, physical capability and enhancing their Quality of Life (QoL) is described below.

## Results

### Insights from the French landscape

Patients receiving treatment for metastatic breast cancer (N = 132), and oncologists specializing in the treatment of metastatic breast cancer (N = 43), were recruited via social media and patient associations or professional organizations, respectively. The demographics of these two populations are shown in **Table 1**. It should be noted that while metastatic and advanced breast cancer are not necessarily equivalent, for the purposes of this survey we are considering them to be so.

**Table 1:** Demographics of participants in the quantitative mirror study (patients & oncologists).

Patients		Oncologists	
Characteristics			
n	132	n	43
Age years median (range)	50 (25 to >75)	Age years median (range)	47 (<45 to ≥55)
Gender	Female 100%	Gender	Male 56%
Treated at a healthcare center	82%		
Treated at home	36%		
Average BMI	25.7		
Physical activity (sport) ≥1x/week	61%		
Evolution of localized tumor	58%		
Activity			
**Not Employed, active**	**39%**	**Hospital based**	**93%**
AB-	10%	Cancer centre	50%
AB+	29%	Clinic, private hospital	3%
**Inactive**	**61%**	General hospital centre	18%
		University hospital centre	8%
		Public assistance	3%
		**Mixed activity**	**7%**
Time since diagnosis (years)			
< 1	18%		
Between 1 and 2	32%		
Between 3 and 4	30%		
≥ 5	20%		
Treatment			
Chemotherapy	75%		
Targeted therapy	72%		
Hormone therapy	61%		
Radiotherapy	57%		
Surgery	44%		
Immunotherapy	25%		

Key features of the two populations who responded to the online questionnaire; patients with metastatic breast cancer, all of whom were suffering from fatigue, and a group of oncologists specializing in the treatment of metastatic breast cancer. NB: the groups are not paired; patients were not asked to disclose where they were treated nor physicians where they worked.

AB+/AB- designed standard social category.

The values in bold may, if necessary, be further detailed into sub-groups.

### Awareness and understanding of CRF

**Figure 1:**
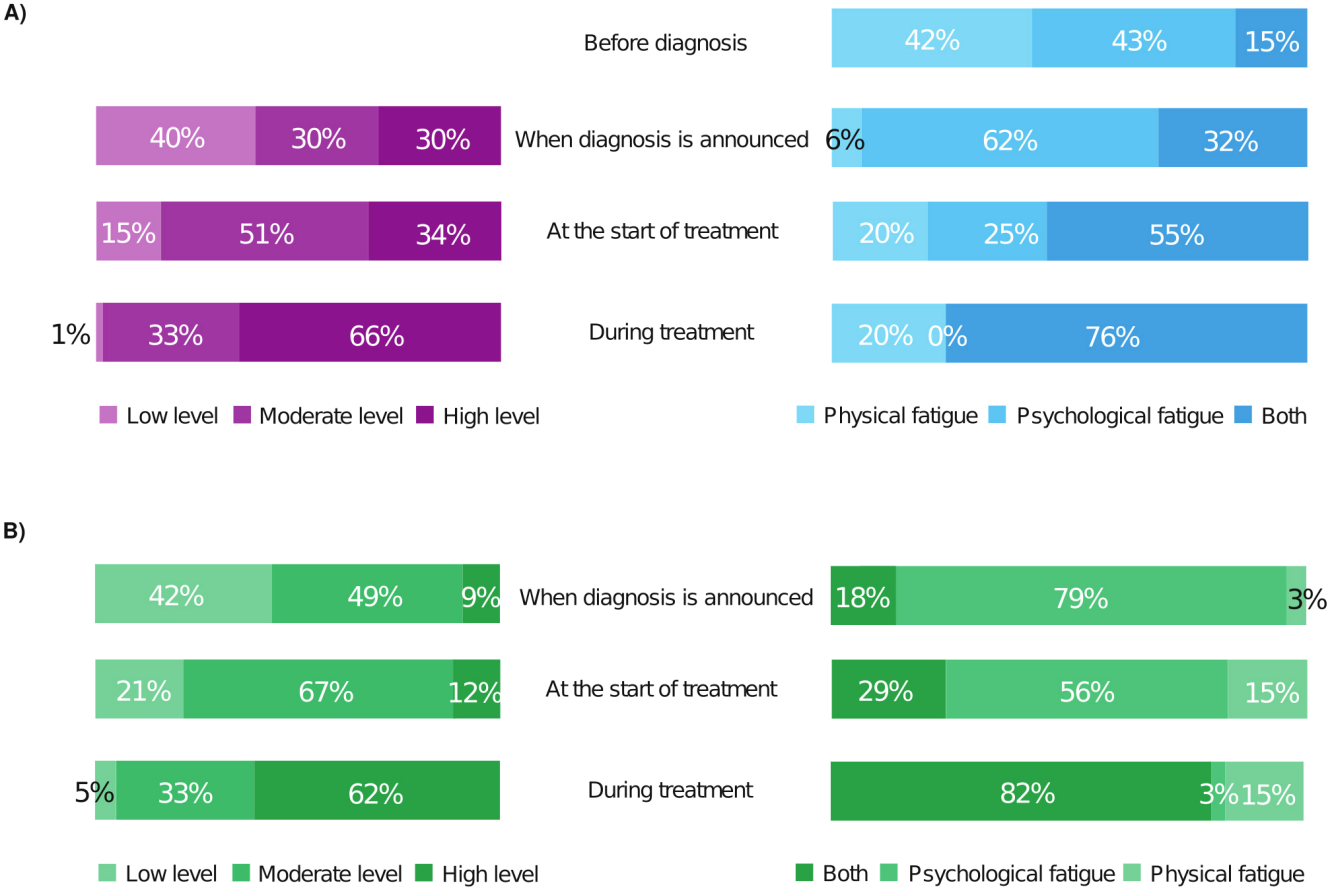
Patients’ **(A)** and oncologists’ **(B)** views differ as to when fatigue becomes problematic, its intensity and type early in the treatment pathway. **(A)** Patients’ answers to single choice questions left panel: “*What was your level of fatigue at each stage of the treatment process?*” (132 replies) Right panel: “*When is fatigue most difficult to deal with?*” (105 replies). **(B)** Oncologists’ responses to single choice questions: right panel “*When is physical and/or psychological fatigue most difficult for your patients with metastatic breast cancer?*” (43 responses). Left panel “*What is the level of fatigue in your patients with metastatic breast cancer at each stage of the care journey?*” (34 responses) Physicians underestimate the intensity of fatigue patients suffer before treatment begins. This includes at the diagnosis of metastatic breast cancer. Oncologists consider psychological fatigue at diagnosis and when treatment starts to be predominant whereas, patients consider there to be a strong physical component. Once treatment is underway their answers converge for both questions.

Both patients (80%) and oncologists (79%), **Figures 1A, B**, respectively, distinguish between psychological and physical fatigue and acknowledged the presence of both in patients with metastatic breast cancer. However, as shown in **Figure 1A**, 30% of patients already suffer high intensity fatigue at the time of diagnosis and 34% before the onset of treatment while physicians consider this to be the case for just 9 and 12% of their patients, respectively. Oncologists consider treatment initiation as an overriding reason for psychological fatigue (79%) whereas 32% of patients consider there is also a physical component to their fatigue. Those in employment at the time of treatment initiation complain of psychological fatigue, whereas those not actively employed are more likely to complain of physical fatigue.

### Impact of CRF on patients’ daily lives

**Figure 2:**
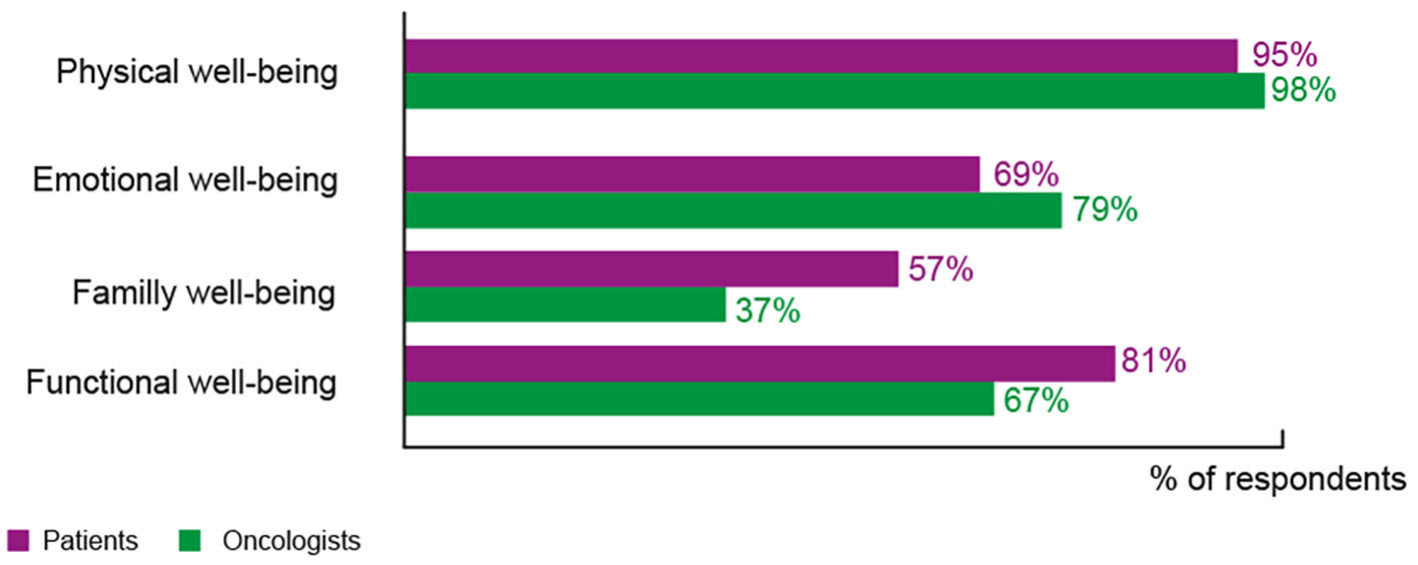
Impact of fatigue on patients’ daily lives. Responses to, Patients’ question: “Here are several elements related to well-being that may have been impacted by fatigue related to your cancer. Can you rank the 5 items that impact you the most?” (132 responses), Doctors’ question: “Here are several elements related to well-being that can be impacted by cancer-related fatigue. Can you classify the 5 major items which, according to you, are impacted by fatigue in your patients?” (43 responses). The details of elements within each domain are given in **Supplementary Table S1**.

Patients and oncologists were asked to rank the five items that fatigue influenced most in their daily lives (mirrored question), the results were then re-grouped under four main categories (**Figure 2**; **Supplementary Table S1**). For 61% of patients, fatigue worsened other treatment side effects, 74% of physicians also thought this, while 59% of patients complained they were lacking energy, only 40% of oncologists considered this to be the case. Fatigue negatively impacts both professional and domestic activities (50%) and affects patients’ sleeping patterns (49%) For 57% of patients, family well-being was adversely affected but only 37% of oncologists thought this aspect would be greatly influenced. Oncologists considered the effect of fatigue on the patient’s intimate life to be only relevant for 7% of cases while 33% of patients stated it was important. Oncologists (40%) considered that fatigue would impact the patient’s morale in regard to their disease compared with 24% of patients.

### Discussion and assessment of CRF

Almost all patients (96%) had discussed fatigue with their oncologist and 66% with their general practitioner. However, only 36% of patients thought that their oncologist took into account their opinions and feelings regarding fatigue. Despite the recognized importance of the role of the oncology nurse in the ABC treatment pathway (27–29), only 36% of patients discussed their fatigue with a nurse. Oncologists (81%) assess a patient’s level of fatigue through questioning during the consultation and observing the patient’s behavior (49%). Only 28% utilize blood markers for further assessment and differential diagnosis, while 21% employ adapted visual analog scales for the evaluation of CRF and 5% use a paper questionnaire.

### Treating CRF and role of the oncologist

**Figure 3:**
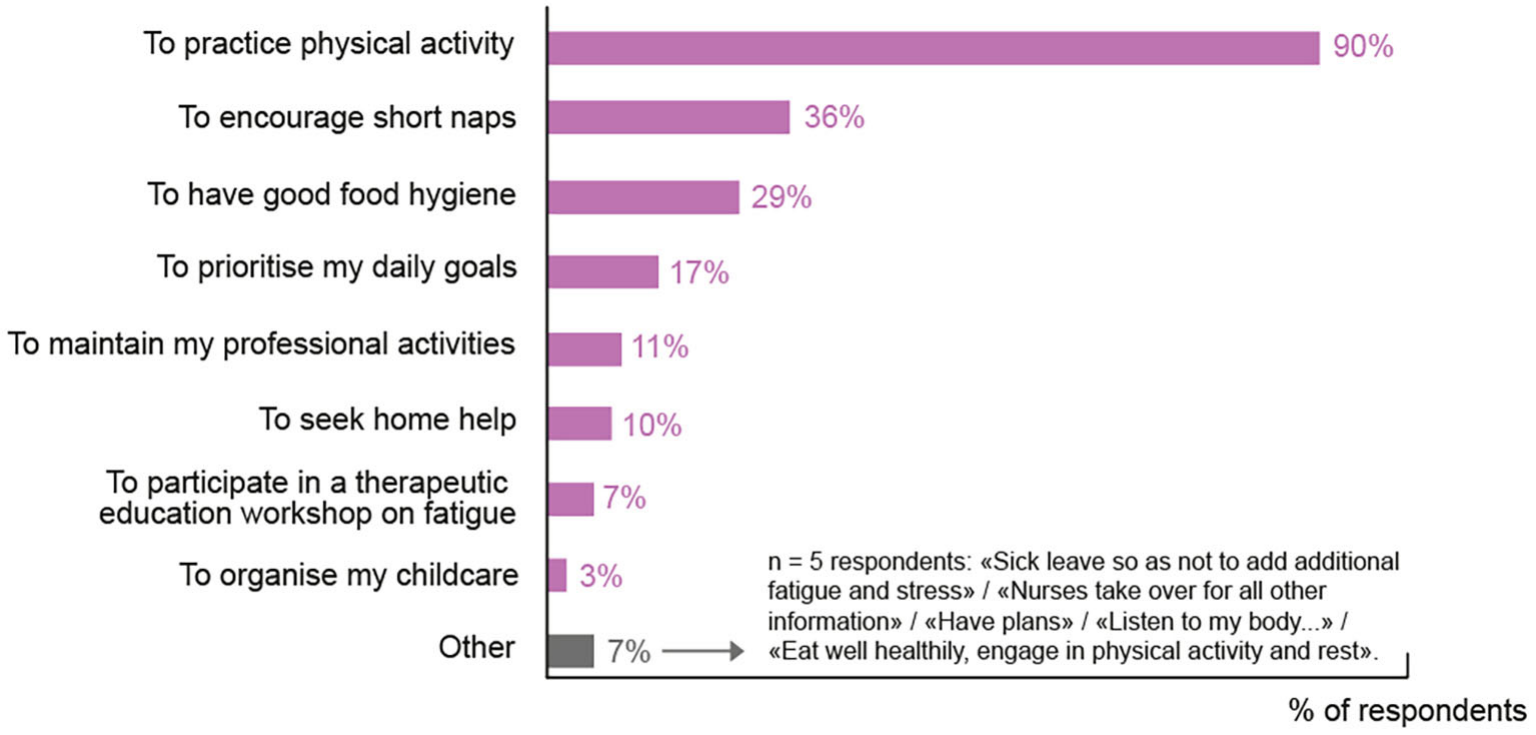
Non-pharmacological solutions proposed to patients to ameliorate CRF. In the survey, 55% of patients agreed that they had received advice as to how to relieve their fatigue themselves. The responses to the question: “*What does the oncologist recommend to help you relieve the fatigue linked to your metastatic breast cancer on your own?*” are shown here (multiple choice question: 132 patients – 72 received at least 1 recommendation).

Just over half of the patients (55%), claimed to have received advice from their oncologist on how to manage their fatigue themselves. In 90% of cases, the recommendation was to have PA (**Figure 3**). However, when asked whether the oncologist had a role to play in the CTRF management, 98% of doctors felt they have a role but only 79% of patients thought the same. When oncologists were asked how they perceived their role, 52% said it was screening for treatable causes and intensity of fatigue in order to propose solutions, 40% felt it was to motivate/direct patients toward supportive care especially adapted physical activity (APA) 26% considered their role to be that of a listener, providing support and advice, and 12% were concerned by the need to adapt treatment in order to manage fatigue. All physicians claimed to have suggested alternative non-medical support, including APA (84%) to their patients. However, only 59% of the patients reported being offered from supportive care, with 51% specifically participating in APA programs. APA is the practice of modifying physical activities, exercises, or sports to meet the needs of individuals with varying abilities, especially those with disabilities or special needs.

All the oncologists (100%) reported that they had no alternative but to adjust the dose of cancer treatment to manage asthenia on at least one occasion in their professional life. Among participating patients, 33% reported a reduction in the dose of their treatment to alleviate fatigue.

### Improving the management of CRF

When offered a choice of practices to improve the management of CRF, oncologists recognized four areas where change could have a positive impact: further educational and professional training in CTRF was identified by 65%, extra time in a consultation with at least half an hour to discuss CRF (47%), provision of a standardized evaluation tool for fatigue, accompanied by clear recommendations to systematically guide management of asthenia at each step of the patient pathway (40%) and expansion of the role of cancer nurses (40%).

These elements were mirrored by the patients’ answers to the open question “*what would be your main expectations regarding the management of your fatigue related to your metastatic breast cancer?*” The most important changes cited were: healthcare professionals listening to them allowing more time to discuss fatigue (30%), having a systematic management pathway to follow (23%) and being given more solid, practical guidance (21%). Patient associations were cited by 36% of patients as playing a role in the management of CRF.

### Experience of the CRF care pathway

The patients’ perspective on the overall care pathway for metastatic breast cancer, on a numerical scale, produced an average score of 6.3/10 compared with 5.6/10 for oncologists based on their experience (numerical scale from 0 to 10/10, 0 being “the experience of care is very bad” and 10 being “the experience of care is very good”). When specifically questioned about the care pathway for fatigue, the average ratings were 3.5/10 for patients and 3.9/10 for physicians, respectively. In both groups, satisfaction regarding CRF management was low, with only 37% of oncologists and 27% of patients considering fatigue management to be satisfactory. Patients, in general, expressed satisfaction with the management of their ABC, with the exception of fatigue.

## Discussion

CRF is a well-recognized symptom of ABC, its intensity and impact on a patient’s daily life may frequently be exacerbated by cancer treatments. This specific manifestation of fatigue, known as CTRF coincides with treatment onset and may last over and beyond its course, in some cases for several years (5). Poorly understood and characterized by the inability of rest, or sleep, to provide relief, improvement of its management might be expected to improve the treatment experience for patients with ABC as a complement to the greater OS and PFS, they nowadays regularly benefit from. Despite existing general recommendations for managing CRF (3, 7, 11), everyday management of the specific manifestation of CTRF is today suboptimal, as our survey confirms. Conscious of the need to actively explore innovative ways to improve the management of CTRF, the Working Group aims to propose an expert opinion of actions that will assist the practical management of CTRF and enable patients to experience a more satisfactory treatment pathway.

The fatigue experienced in cancer is of two types, psychological and physical. In the survey, doctors were shown to overestimate the importance of psychological fatigue at the time of diagnosis and before treatment unlike patients, who consider that physical fatigue is already present at this point. This divergence is of potential importance as it is known that if a symptom is present before treatment begins, it will only worsen, often significantly, once therapy is initiated (30). However, CRF may diminish with the effectiveness of the treatment in a certain number of cases, despite the fatigue inherent in the anti-cancer treatment. The patients’ impression of fatigue differs at treatment initiation depending on their employment status. Those in employment at the time of treatment initiation complain of psychological fatigue whereas those not actively employed are more likely to complain of physical fatigue. Once treatment begins however, the opinions of both groups converged, with the physical effects of therapy causing fatigue mutually recognized. Therefore, it is crucial to establish a precise diagnosis to exclude any alternative causes of fatigue beyond the impact of treatment.

CRF has a significant impact on many different areas of life which emphasizes its multifactorial nature. When the perceptions of patients and medical doctors on the dimensions affected by fatigue are compared, discordance becomes apparent. Oncologists seem mainly focused on the patient’s “physical” well-being, and less on the “social” side. While more than half of patients feel that fatigue adversely affects family well-being, just over a third of oncologists are of this opinion. The effect on a patient’s intimate life is also underestimated by oncologists despite a third of patients stating that their intimate life is affected by fatigue. On the other hand, doctors overestimate the effect of fatigue on the patients’ hope to overcome their disease.

Addressing the issues of fatigue linked to treatment is crucial and requires open discussions within the medical field and with patients to optimize treatment outcomes. The survey shows that discussion of fatigue with oncologists is both possible and desired by patients underscoring a willingness to engage in conversations about fatigue. However, there were discrepancies in the answers that show this is an area where improvement is required. Firstly, the majority of oncologists stated that they assess fatigue levels at consultation but the low response rate from patients (n=8/132) on this item suggests that the patient themselves may not be aware, or do not know whether, their doctor is assessing their fatigue. This reinforces the need for better communication between doctor and patient regarding fatigue characterization. Moreover, the patients strongly felt that when it came to fatigue, they were not being listened to, and healthcare professionals exhibited a sense of “*fatality*” when the subject was raised (33%), an adverse event that had to be “*lived with*”. While oncologists are aware of these patient concerns, they feel that the dedicated structure needed to support patients is missing. Considering the recognized importance of their role and their proximity to the patient (27–29), surprisingly few patients had discussed their fatigue with their cancer nurse. This is suggestive of potential gaps in the actual implementation of care.

The improvement of CTRF management is also crucial to prevent the necessity to reduce the dose of anticancer treatment. Dose reduction is associated with poorer overall survival (31). Every oncologist surveyed reported that they had, at least at one point, needed to reduce therapeutic doses, suggesting that CTRF can be so severe that oncologists often feel they have no alternative options. There is clear evidence that CTRF can be ameliorated by APA which reduces insomnia and improves the quality and quantity of sleep (30). Additionally, it increases cardio-respiratory fitness, with an improvement in inflammatory variables. Adapted exercise is safe and well tolerated by patients undergoing cancer treatments or in the rehabilitation phase and offers patients a non-pharmacologic alternative which is appealing (7, 11, 16, 21). Consistent with current best practice guidelines, the majority of surveyed oncologists said they recommended APA to their patients, while only half the patients who responded had participated in APA programs. These results may highlight potential miscommunication between patient and physician or a broader inability to implement APA, indicating that adequate time and support mechanisms may not be in place. Oncologists in our survey mentioned the lack of sports facilities to offer and the lack of dedicated time in consultations to accompany patients further in their care, which coupled with a deficit of therapeutic options, creates an important unmet need in the management of CTRF. These findings are similar to those of a larger nationwide study carried out by AFSOS (*Association Francophone Des Soins Oncologiques De Support*) in 2013 which found that among 700 doctors and 1–500 patients, 98% of oncologists said they had offered advice but only 55% of patients had any recollection of supportive care being proposed (32). The AFSOS 2017 report suggests, once again, that communication about the available support services is not reaching patients and is offered late in the treatment process (33).

Even when offered, in practice there are considerable barriers to its effective implementation including the need for a solid network of sports centers and coaches to maintain the motivation required by patients to obtain positive effects. There is also a lack of evidence as to the best exercise routine to follow. While many systematic reviews with meta-analyses on exercise and CRF have been conducted, the direction of results and especially the magnitude of effect have varied substantially (19, 30, 31, 34). Thus, leaving physicians with the knowledge that APA should be offered but unsure as to the best program to recommend.

A major finding from this new survey was that more time should be committed to understanding CTRF. Oncologists were of the view that training would strengthen their knowledge base and allow them to offer more practical advice to their patients, citing a lack of clinical research and a need for effective treatments. For successful management, frequent reiteration is necessary. To implement this effectively and to avoid over burdening already busy personnel, certain tasks (announcement consultation, complete fatigue assessment, access to supportive care etc.) could be delegated to other HCPs particularly oncology nurses in order to create a seamless and effective pathway. Patients surveyed referred frequently, to having obtained help with their fatigue from sources other than their oncologist.

From recent systemic reviews and studies it would appear that interventions to combat CRF in cancer patients and survivors led by nurses show particular promise. This is especially the case if the focus is multidisciplinary including, exercise and psychological, and behavioral approaches (35). More research is needed in order to determine the most acceptable forms of intervention, but it appears that even relatively short periods of nurse-led exercise intervention, 4 to 6 weeks, can have positive effects (36). If such interventions also adopt a holistic approach to care they are particularly effective in improving health-related quality of life if (37). These findings are aligned with those of the survey in which 40% of the oncologists thought that expanding the role of the oncology nurse would be beneficial to outcomes.

Patients also obtain help from their GPs and patient associations. The latter provide valuable support by facilitating the sharing of experiences, offering supportive oncological care, and presenting complementary alternative practices. A collaborative approach between physicians and these associations could be constructive to ensure that patients receive well-informed guidance and holistic support in addressing CRF. Multidisciplinary commitment and communication are primordial to successful outcomes, which extends to possible external sources for management of APA.

Finally, the lack of oversight from the oncologist regarding the validity and way patients are treated outside the healthcare establishment is a notable concern. To address this, patient associations could play a crucial role by organizing informative sessions on recommended alternative practices, such as webinars, providing education to patients. Digital tools, particularly exercise apps, can also be used to enhance patients’ knowledge and provide them with personalized advice.

### Potential limitations

The survey conducted in this study was limited to France, and the findings may not be directly applicable to other European countries. Additionally, the specific care settings of the patients were not determined, introducing a potential variability in healthcare contexts. No specific profile requirements for participants, other than those stated above were applied to either group. It should be noted that the patients and physicians were not paired, which means comments are not applicable to particular cases but to perceptions in general. The use of social media for recruitment may have introduced bias, as reflected in the younger age of the patient cohort (median 50 years), considering that a significant proportion of breast cancer cases in Europe occur in patients older than 50 (21). This age difference could contribute to variations in opinions on domains affected by fatigue or suggest potential differences in the areas freely discussed with oncologists. Additionally, the recruitment through patient associations and medical societies focused on supportive care in oncology may have led to respondents who are particularly informed and interested in the topic of fatigue.

## Takeaways

CTRF has a substantial impact on quality of life, particularly on well-being, for patients with ABC. However, CTRF is not an inevitability and its effects can be mitigated through careful management, including the differential diagnosis from CRF. Once diagnosed immediate adjustment of therapeutic doses, especially discontinuation of treatment, is not recommended. Dose reduction of anticancer therapies should be considered an exceptional measure and a last resort. APA should be offered to all patients, starting before the initiation of treatment and continuing throughout its course, as it offers physiological, physical, and psychological benefits to all patients. The management of CTRF requires a multidisciplinary approach that extends from the hospital into the wider healthcare community.

## Expert opinion

**Figure 4:**
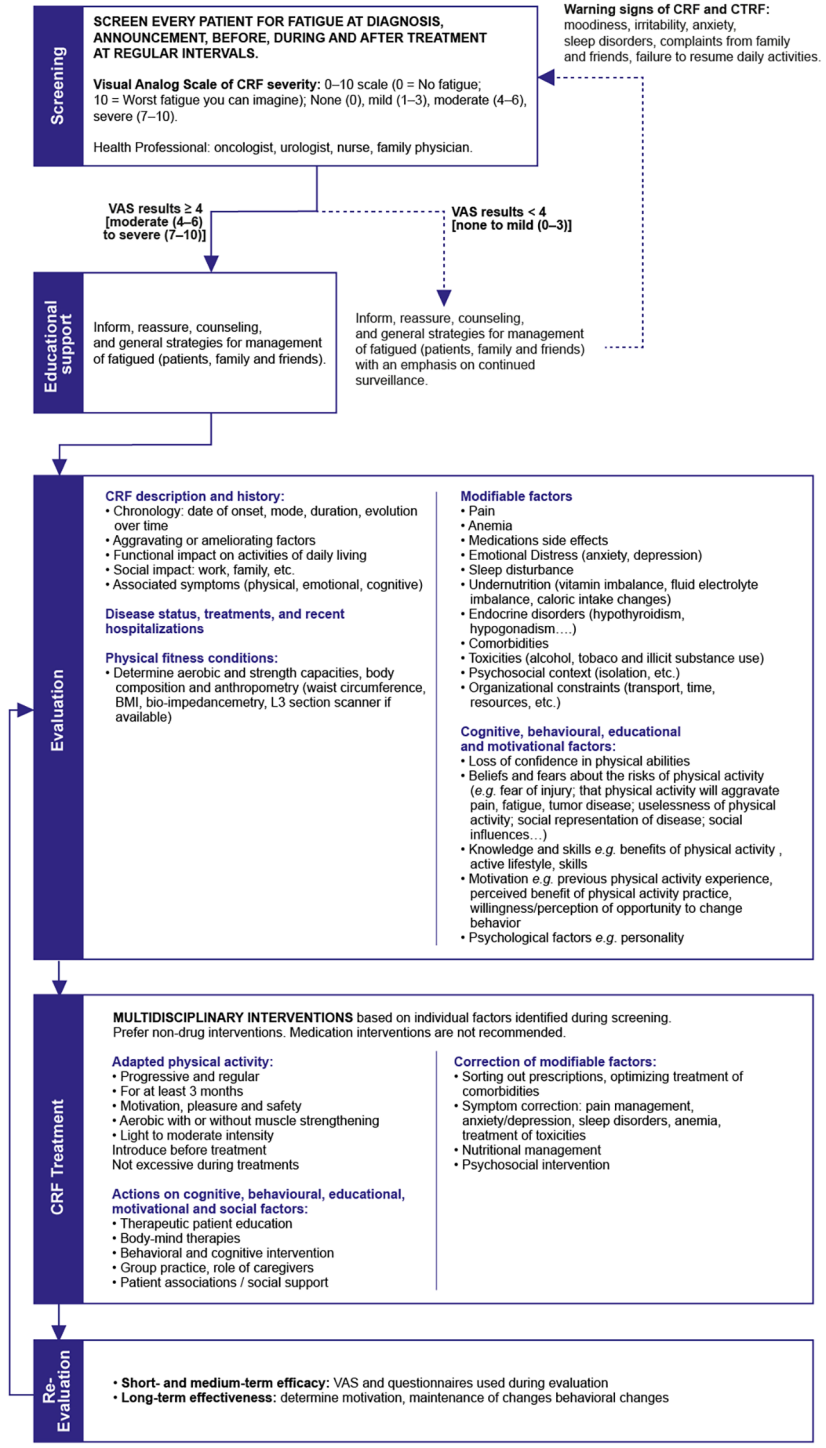
CRF management algorithm. Adapted from ESMO 2020, AFSOS 2021 and NCCN 2023 (4, 8, 11).

To provide a comprehensive and patient-centered care approach for CTRF, the Working Group has elaborated five key strategies to ensure the prompt identification and effective management of CTRF in ABC patients (**Table 2**). These suggestions are intended to support oncologists in the diagnosis and assessment of fatigue during the treatment process and to elucidate the priorities for CTRF management, drawing upon the expertise of nutritionists, psychiatrists, pain management experts, and supportive care providers (**Figures 4**). It is important to note that these should be customized to account for the capabilities of the medical facility and the specific attributes of individual patients, including comorbidities, concurrent medications, medication history, and unique patient preferences. Treatment decisions should always be made through a mutual agreement involving patients, HCPs and other caregivers.

**Table 2:** Key steps based on expert opinion for the management of CTRF in clinical practice. For full details see text.

Key step	Elements to consider
*1. Screening and assessing CTRF in ABC patients throughout the healthcare pathway*	• Baseline assessment before the initiation of treatment and iterated to diagnose fatigue induced by therapy throughout the course of cancer treatment. • Encourage patient to describe their fatigue and its impact on their daily activities using clear and straightforward language. • Select simple and relevant questions from existing diagnostic questionnaires, such as ‘*Do you feel tired quickly?*’, ‘*When do you feel most tired?*’ *‘Why do you think you’re tired?’* or ‘*Do you have problems remaining focused?* • Ask patient to grade the duration and intensity of their fatigue symptoms and to determine whether these symptoms are associated with treatment administration (Patient reported Outcomes (PRO)-CTCAE). • Consider the social and environmental contributors. CTRF contributing factors include pain, nausea, anemia, sleep disturbances, malnutrition, sarcopenia, physical inactivity, cognitive dysfunction, and emotional distress, including anxiety. Determine their impact over time. • Involve nurses whenever possible.
2. *Counseling and informing about CTRF*	• Deliver comprehensive information to patients regarding the diagnosis of fatigue and the available strategies for its management. • Organize a specialized “announcement consultation” to carry out a shared educational assessment with the patient in order to guide them toward appropriate workshops.
*3. Directing patients to supportive care interventions*	• Guide patients to the most suitable supportive care resources, support groups, and patient associations. • Create a list of local/regional contacts and potential resources (rehabilitation therapists, pain specialists, nutritionists, psychiatrists/psychologists, fitness instructors, social workers, and patient organizations).
4. *Integrate pain, psychological, nutritional and physical assessments and support*	• Prioritization of non-pharmacological approaches, including PA, nutritional support and psychosocial interventions. • Focus on Adapted Physical Activity & Nutritional support • Engage all ABC patients in PA adapted to their ability and circumstances. • Establish local APA prescription service to conduct a comprehensive assessment of fatigue and develop personalized intervention programs for each ABC patient. • Allocate dedicated time for PA within the hospital department at the onset of the therapeutic strategy. • Assure a common message between healthcare providers regarding APA benefits. • CRF and physical assessments may be conducted every 6 months. • Enhance HCP’s capacity to provide appropriate exercise education and rehabilitation (specific guidelines for PA testing and prescription; collaborate with exercise specialists). Participate in one, or more, of the similar cancer rehabilitation programs that are available for exercise professionals specializing in the care of cancer patients.
5: *Eliminate and treat all other possible causes of fatigue before considering anti-cancerous treatment dose reduction.*	• Dose reduction of anticancer treatment should be considered only if necessary.

### Key n°1: screening and assessing CTRF in ABC patients throughout the healthcare pathway

Knowing that CTRF is a subjective experience and, in line with the latest guidelines, the first stage is to comprehensively screen for CTRF through a combination of patient self-assessment and clinical examination (7). Assessments should be performed before the initiation of treatment and iterated to diagnose fatigue induced by therapy throughout the course of cancer treatment (3, 7). Based on expert insights, patients should be encouraged to describe their fatigue and its impact on their daily activities using clear and straightforward language. By asking simple questions about the patient’s physical and psychological well-being, oncologists can inquire whether the patient is experiencing persistent exhaustion associated with CTRF. The physician should make it clear to the patient that they will be conducting an interview to assess fatigue. Questioning will be particularly important with those patients neglecting fatigue or tending to consider it as an unavoidable consequence of cancer (23). Additionally, it is important to note that the fear of cancer progression plays a significant role in the underreporting of fatigue (3).To further assess fatigue symptoms, HCPs may select simple and relevant questions from existing diagnostic questionnaires, such as ‘*Do you feel tired quickly?*’, ‘*When do you feel most tired?*’ *‘Why do you think you’re tired?’* or ‘*Do you have problems remaining focused?*’.

The French National Cancer Institute’s (INCa) questionnaire, provided in the **Supplementary Materials**, is an example of a valuable tool to encourage patient self-assessment during clinical visits (38). This questionnaire consists of 12 questions dedicated to assessing CRF, anxiety, depression, and insomnia. Patients are asked to rate the intensity of each symptom on a scale from 0 (no symptoms) to 4 (frequent). Additionally, they should specify the time of day when these symptoms occur (morning, noon, afternoon, evening, and night). While this questionnaire provides a comprehensive assessment of fatigue and related symptoms, completing it is time-consuming. Physicians often have limited time for fatigue screening due to their clinical workloads, therefore the panel recommends involving nurses whenever possible to actively engage in patient questioning (33).

As part of the CTRF self-assessment, patients should also be encouraged to grade the duration and intensity of their fatigue symptoms and to determine whether these symptoms are associated with treatment administration. To this purpose, fatigue grading may be realized using the 5-point scale released by the National Cancer Institute Common Terminology Criteria for Adverse Events (CTCAE). The companion “Patient reported Outcomes (PRO)-CTCAE” lexicon might be used to enable grading of the symptoms from the perspective of the subject experiencing it e.g. fatigue ranging from “none”, “mild”, “moderate”, “severe” to “very high severity” (22, 39, 40).

The assessment of CTRF should be an ongoing process beginning at baseline and then regularly repeated at least at every treatment cycle.

Following the initial fatigue screening, the following step focuses on the diagnostic assessment of CTRF and its constituent factors. The panel advises a continuous evaluation of CTRF and recommends the assessment of contributing factors that may necessitate treatment. These factors include pain, nausea, anemia, sleep disturbances, malnutrition, sarcopenia, physical inactivity, cognitive dysfunction, and emotional distress, including anxiety (7, 40). The consideration of laboratory tests, as detailed in **Table 3**, is encouraged (7). Importantly, the evaluation of these contributing factors should be an ongoing process to determine their impact on CTRF over time and to assess whether they too are linked to the administration of anticancer therapy. The level of a patient’s fatigue should be assessed before initiating any new anticancer treatment. This pre-treatment assessment allows HCPs to establish a baseline understanding of the patient’s fatigue levels and related symptoms. It provides essential information for tailoring the treatment plan to the patient’s specific needs and addressing any potential issues related to cancer-related fatigue. Furthermore, when evaluating fatigue-related manifestations, it is essential to consider the social and environmental contributors, such as a patient’s need to maintain employment during treatment (7, 11). In this context, the panel recommends tailoring the treatment plan to meet the specific needs of each individual patient.

**Table 3:** Recommended laboratory tests for CTRF diagnostic assessment.

Sample	Test
Urine	Urinalysis for protein, blood and glucose
	Urea and electrolytes
Blood	Full blood count
	Liver function
	Blood glucose
	Dosage of the LDH enzyme (for patients receiving immunotherapy)
	Thyroid function
	C reactive protein
	Anemia assessment (include full iron assessment)
	Endocrine assessment
	Nutritional assessment

### Key n° 2: counseling and informing about CTRF

The second key point focuses on the importance of counseling and educating patients about the etiology, symptoms, and treatments of fatigue to optimize the management of CTRF. In line with the most recent guidelines, oncologists and nurses should deliver comprehensive information to patients regarding the diagnosis of fatigue and the available strategies for its management (3, 7, 16, 21). Providing patients with a better understanding of CTRF may help the patient cope with the challenges of exhaustion. Moreover, by imparting knowledge on fatigue management, educational interventions can empower patients to express their preferences regarding treatment options and encourage active participation in shared decision-making (7). As part of the care pathway for ABC, the Working Group suggests that oncologists organize a specialized “announcement consultation” during which they carry out a shared educational assessment with the patient in order to subsequently guide them toward appropriate workshops. For example, patients might be directed to workshops focused on understanding metastatic disease and treatment objectives.

### Key n° 3: directing patients to supportive care interventions

The third key point acknowledges the importance of guiding patients to the most suitable supportive care resources, support groups, and patient associations (40). Because CTRF should be approached in a multidisciplinary manner, the Working Group advocates for oncologists to play an active role in informing patients about supportive care and facilitating their connection with relevant care providers. These providers may include rehabilitation therapists, pain specialists, nutritionists, psychiatrists/psychologists, fitness instructors, social workers, and patient organizations (40). Whenever feasible and in accordance with available resources, it may be beneficial to provide patients with a list of contacts to facilitate their access to various services. Patients in advanced stages of cancer often face challenges in adhering to behavioral changes and require support from various organizations or patient associations. These play a crucial role in providing assistance and resources to patients as they navigate their cancer journey.

### Key n°4: integrate pain, psychological, nutritional and physical assessments and support

The fourth key point emphasizes the prioritization of non-pharmacological approaches, including PA, nutritional support and psychosocial interventions for symptom management and physical recovery (40).

#### Promote an adapted physical activity

First and foremost, it is essential to underscore that PA is both feasible and safe for patients with cancer at all stages of the disease. Additionally, it is proven to be effective in improving various aspects of patient well-being, including fitness and functional capacity, strength, quality of life, and fatigue (20). There is strong evidence that PA has an immune-stimulating effect, mobilizing immune cells that may target tumors within hours after exercise. Tailoring exercise programs to each patient can have a positive impact on their health status (18). Therefore, the Working Group strongly recommends that all ABC patients should actively engage in PA adapted to their ability and circumstances. The minimum PA should be 30 minutes/day, 3 days/week for at least 6 months with the objective of continuing the regimen even on treatment completion (41).

Although APA is beneficial for all patients, adherence rates vary depending on individual attitudes and experience prior to the cancer diagnosis. Some patients may have prior experience while others may be resistant to PA but discover its benefits during treatment (42). To enhance patient adherence to PA programs, HCPs should assist patients in connecting with qualified trainers and accessible fitness centers, as the inaccessibility of such facilities is often perceived as a barrier to participation (22).

For an optimized APA program, it is important to integrate psychological, nutritional, and physical assessments. The Working Group suggests allocating dedicated time for PA within the hospital department at the onset of the therapeutic strategy. This would entail distributing responsibilities among various stakeholders, including local patient associations, nurses, nutritionists, psychologists, physiotherapists, and oncologists: each participant contributes to reinforcing the common message while providing their unique perspective. The Working Group proposes to establish a local APA prescription service to conduct a comprehensive assessment of fatigue and develop personalized intervention programs for each ABC patient. The assessment is carried out as part of an outpatient program involving a multidisciplinary team consisting of, at least, an oncologist, a physiotherapist, and an APA coach. This pathway serves multiple purposes: 1) verification of the absence of contraindications to APA, 2) assessment of the patient’s general physical condition, 3) evaluation of the patient’s motivation and geographical and financial availability to participate in APA programs. CRF and physical assessments may be conducted every 6 months by the healthcare team. Additionally, this service can help patients who have difficulty traveling to the healthcare facility, connect with local associations or exercise coaches, enabling them to regularly follow their APA programs.

Finally, given the essential role of the oncologist in promoting patients’ engagement in PA (22), they are encouraged to enhance their capacity to provide appropriate exercise education and rehabilitation. To this purpose, oncologists should refer to specific guidelines for PA testing and prescription and collaborate with exercise specialists, such as physiotherapists and rehabilitation experts, for the assessment and prescription of exercise (3). Before recommending a particular training program, oncologists are advised to participate in one, or more, of the similar cancer rehabilitation programs that are available for exercise professionals specializing in the care of cancer patients.

#### Nutritional support

Patients with ABC are generally advised to adopt a well-balanced dietary regimen incorporating vegetables, fruits, whole grains, legumes, protein sources, and sufficient hydration (a minimum of 2 liters daily). Importantly, current guidelines recommend the formulation of a dietary plan in collaboration with a registered dietitian prior to the onset of symptoms (40). The relationship between a patient’s nutrition and their ability to engage in APA is reciprocal. Improved nutrition enhances the patient’s capacity to participate in APA, and in turn, engaging in APA can promote better nutritional habits. This symbiotic relationship underscores the importance of addressing both nutrition and PA to optimize the overall well-being of ABC patients.

#### Consideration of pain

The presence of pain can negatively impact adherence to APA. Therefore, the assessment of pain should be an integral part of every medical consultation. It is helpful to have a comprehensive plan for pain management throughout the ABC care pathway in place. This may include both pharmacological and non-pharmacological interventions, and it is crucial that patients fully understand and engage with the proposed strategies (3).

#### Actions on anxiety, depression, and insomnia

The Working Group considers referring patients to mental health professionals to evaluate the need for pharmacological interventions aimed at reducing anxiety or improving sleep.

### Key 5: eliminate and treat all other possible causes of fatigue before considering treatment dose reduction

The primary objective of CTRF management is to enhance the quality of life for patients without impeding the management of their disease by discontinuing treatment (16, 21). This consideration is particularly important for elderly ABC patients, for whom CTRF therapy should be finely tuned to preserve functional independence.

Given that cancer treatment itself can be a causal factor of fatigue (3, 7), experts unanimously advocate elimination of all other possible causes of fatigue before consideration of dose reduction. If the fatigue is attributed to the treatment, implementing supportive care measures such as APA should be prioritized, with dose reduction considered only if necessary.
