# Supplementary material for: Management of cancer treatment-related fatigue in advanced breast cancer patients: an expert committee’s opinion
Source: Front Oncol. 2025 Oct 9;15:1617600. doi: 10.3389/fonc.2025.1617600 (PMC12545012; doi:10.3389/fonc.2025.1617600)
Supplement: Supplementary file 1 [file DataSheet1.pdf]

SUPPLEMENTARY APPENDIX

Supplementary appendix 1 - Survey questionnaire (in French). Patient.

QCU: Single-choice question; QCM: Multiple-choice question; QO: Open question; QNUM: Numerical question; QCLASSEMENT: Classification question ; Mandatory answer to each question. Questionnaire with no backspace.

SCREENER & ETAT DES LIEUX DE LA MALADIE

Q1. Etes-vous ou avez-vous été personnellement concerné.e par un cancer du sein métastatique ? QCU. 1. Oui, j'ai passé des examens et j'attends un diagnostic (STOP INTER) ; 2. Oui, on m'a diagnostiqué un cancer du sein métastatique et je ne suis pas de traitement ou n'ai pas encore commencé mon traitement (STOP INTER) ; 3. Oui et je suis en cours de traitement ; 4. Oui, je suis actuellement en pause ou en arrêt de traitement (STOP INTER) ; 5. Non, je n'ai jamais eu un cancer au stade métastatique) (STOP INTER). STOP INTER : *afficher le message de remerciement « Nous vous remercions pour votre participation. Malheureusement, vous ne correspondez pas au profil que nous recherchons »*

Q2. Est-ce que votre cancer actuel est une récidive d'un cancer localisé ou est-il métastatique d'emblée ? QCU. 1. Oui, ma maladie est une évolution d'un cancer localisé. 2. Non, ma maladie n'est pas une évolution d'un cancer localisé.

Q3. Quelle est l'ancienneté de votre diagnostic du cancer du sein métastatique ? QCU. 1. Moins d'un an ; 2. Entre 1 et 2 ans ; 3. Entre 3 et 4 ans ; 4. 5 ans et plus.

Q4. Quels sont ou quels ont été vos traitements pour soigner votre cancer du sein métastatique ? QCM sauf code 7 exclusif. 1. Chirurgie ; 2. Chimiothérapie ; 3. Radiothérapie ; 4. Hormonothérapie ; 5. Thérapies ciblées ; 6. Immunothérapie ; 7. Je ne sais pas.

Q5. Parmi votre ou vos traitements pour soigner votre cancer du sein métastatique, avez-vous reçu ENHERTU (trastuzumab déruxtecan) ? QCU. 1. Oui ; 2. Non ; 3. Je ne sais pas.

Q6. Où se sont déroulées vos séances de traitements ? QCM. 1. En établissement de soins, avec hospitalisation ; 2. En établissement de soins, en hôpital de jour ; 3. A votre domicile en HAD (Hospitalisation A Domicile) ; 4. A votre domicile, sans HAD (Hospitalisation A Domicile).

VECU ET IDENTIFICATION DE LA FATIGUE

Q7. Pouvez-vous indiquer, sur une échelle de 0 à 10, comment vivez-vous votre parcours de soins dans le cadre de la prise en charge de votre cancer du sein métastatique ? MIROIR. *QNUM* **(**0 = le parcours de soins est très mal vécu et 10 = le parcours de soins est très bien vécu)

Q8. Distinguez-vous ce qui relève de la fatigue physique et ce qui relève de la fatigue psychologique ? MIROIR. *QCU* 1. Oui ; 2. Non

Q9. Pour chacune des étapes du parcours de soins, quel a été votre niveau de fatigue ? MIROIR. *QCU – une seule réponse possible par ligne.*

	Niveau de fatigue	Niveau de fatigue	Niveau de fatigue

	faible	modéré	élevé
1. A l’annonce du diagnostic	1	2	3
2. Au démarrage du traitement	1	2	3
3. Pendant le traitement	1	2	3

À ceux qui distinguent ce qui relève de la fatigue physique de la fatigue psychologique : **Q10. A quel(s) moment(s) la fatigue physique et/ ou la fatigue psychologique sont les plus difficiles à vivre ?** MIROIR. *QCU – une seule réponse possible par ligne*

	Fatigue physique	Fatigue psychologique	Les deux
1. Avant le diagnostic	1	2	3
2. A l’annonce du diagnostic	1	2	3
3. Au démarrage du traitement	1	2	3
4. Pendant le traitement	1	2	3

Q11. Voici une liste de caractéristiques pouvant expliquer la fatigue, pour le plus grand nombre. Pouvez-vous classer par ordre décroissant les 5 principales causes de la fatigue que vous ressentez ou avez ressenties ? MIROIR. *QCLASSEMENT - en 1er, en 2e, en 3e, en 4e et en 5e – rotation aléatoire des items sauf code 15.* 1. Progression de la maladie ; 2. Les réactions de défense de mon corps ; 3. Les traitements contre le cancer ; 4. Les variations de mon poids ou de ma masse musculaire ; 5. Les douleurs ; 6. L'anxiété ; 7. La dépression ; 8. L'anémie ; 9. Les troubles de mon sommeil ; 10. Les difficultés pour m'alimenter ; 11. Le fait de me sentir amoindrie et vulnérable ; 12. La réduction de mon activité physique ; 13. L’augmentation de ma sédentarité ; 14. Les contraintes organisationnelles (déplacements pour les soins (distance et durée), fréquence des consultations) ; 15. Une autre raison.

Q12. Avez-vous déjà parlé de votre fatigue liée à votre cancer du sein métastatique, à un professionnel de santé ? MIROIR. *QCU* 1. Oui ; 2. Non.

À ceux qui ont déjà parlé de leur fatigue à un PS : **Q13. Avec quel(s) professionnel(s) de santé avez-vous parlé de votre fatigue en lien avec votre cancer du sein métastatique ?** *QCM ;* 1. Mon oncologue ; 2. Mon médecin traitant ; 3. Mon pharmacien ; 4. Mon kinésithérapeute ; 5. L’infirmière d’hôpital de jour ou d’hospitalisation à domicile ; 6. Un autre professionnel de santé (*question ouverte*).

À ceux qui n’ont jamais parlé de leur fatigue à un PS : **Q14. Pour quelle(s) raison(s) n’avez-vous jamais parlé de votre fatigue en lien avec votre cancer du sein métastatique à un professionnel de santé ?** *QO.*

Q15. Votre bien-être a été impacté par la fatigue. Nous avons regroupé 4 catégories de bien-être et leur avons associé à des items. Selon vous, sélectionnez et classez les 5

items majeurs, toutes catégories confondues, qui vous impactent ? MIROIR. *QCLASSEMENT- en 1er, en 2e, en 3e, en 4e et en 5e – rotation aléatoire des items au sein de chaque catégorie.* 1. LE BIEN ÊTRE PHYSIQUE : a. Manque d'énergie ; b. Nausées ; c. Incapacité à assumer son rôle dans la famille ; d. Douleurs ; e. Effets secondaires du traitement ; f. Forte sensation d'être malade ; g. Alitement.

2. Le BIEN ÊTRE EMOTIONNEL a. Tristesse ; b. Insatisfaction de la façon dont il gère sa maladie ; c. Perte d'espoir dans la lutte contre la maladie ; d. Nervosité ; e. Appréhension de la dégradation de son état. 3. LE BIEN ÊTRE SOCIAL ET FAMILIAL a. Eloignement des amis ; b. Absence ou manque de soutien amical et familial ; c. Déni de la maladie par la famille ; d. Eloignement du partenaire ; e. Incidence sur sa vie intime ; f. L'isolement. 4. LE BIEN ÊTRE FONCTIONNEL a. Impact sur ses activités professionnelles et domestiques ; b. Rejet du travail ; c. Altération du sommeil ; d. Déni de la maladie par le patient.e.s ; e. Désintérêt pour les loisirs.

PRISE EN CHARGE DE LA FATIGUE

Q16. Sur une échelle de 0 à 10, comment qualifieriez-vous l'efficacité de la prise en charge de votre fatigue liée à votre cancer du sein métastatique ? MIROIR. (Sachant que 0 = pas du tout satisfaisante et 10 = très satisfaisante) *QNUM* --/10

Q17. Votre médecin vous a-t-il proposé une évaluation de votre fatigue en lien avec votre cancer du sein métastatique ? MIROIR. *QCU ;* 1. Oui, spontanément ; 2. Oui, suite à ma demande ; 3. Oui, suite à un bilan ou/et une consultation ; 4. Non.

A ceux qui ont eu une proposition d'évaluation de leur fatigue : **Q18. Comment le médecin a effectué l'évaluation de votre fatigue liée à votre cancer du sein métastatique ?** MIROIR. *QCM.* 1. Via des questions orales posées en consultation ; 2. Via une réglette ; 3. Par l'observation de votre comportement ; 4. Via un questionnaire papier ; 5. Avec une tablette ; 6. Via un bilan biologique et clinique ; 7. Autre *(question ouverte).*

Q19. Que vous recommande l'oncologue pour vous aider à soulager par vous-même votre fatigue liée à votre cancer du sein métastatique ? *QCM.* 1. De favoriser des siestes courtes ; 2. D'avoir une bonne 3ygiene alimentaire ; 3. De conserver mes activités professionnelles ; 4. De pratiquer une activité physique ; 5. De me rapprocher des aides à domicile ; 6. D'organiser la garde de mes enfants ; 7. De hiérarchiser mes objectifs journaliers ; 8. De participer à un atelier d'éducation thérapeutique sur la fatigue ; 9. Autre *(question ouverte).*

Q20. Diriez-vous que votre oncologue a un rôle à jouer dans la prise en charge de votre fatigue en lien avec votre cancer du sein métastatique ? MIROIR. *QCU.* 1. Oui, tout à fait ; 2. Oui, plutôt ; 3. Non, plutôt pas ; 4. Non, pas du tout.

Q21. D'autres professionnels ont-ils joué un rôle dans la prise en charge de votre fatigue ? *QCM sauf code 9 exclusif.* 1. Infirmière hospitalière ; 2. Infirmière en ville ; 3. Pharmacien ; 4. Médecin généraliste ; 5. Rééducateur sportif ; 6. Kinésithérapeute ; 7. Association ; 8. Un autre professionnel *(question ouverte) ;* 9. Non, aucun autre professionnel.

Q22. Et diriez-vous que votre oncologue prend suffisamment en compte votre avis et vos sensations concernant votre fatigue ? *QCU.* 1. Oui, tout à fait ; 2. Oui, plutôt ; 3. Non, plutôt pas ; 4. Non, pas du tout.

Q23. Est-ce que votre médecin a déjà modifié ou ajusté votre traitement anticancéreux en vue de diminuer votre niveau de fatigue ? MIROIR. *QCU.* 1. Oui, au point de l'arrêter ; 2. Oui, régulièrement ; 3. Oui, quelques fois ; 4. Oui, rarement ; 5. Non, jamais ; 6. Je ne sais pas.

Q24. Votre oncologue vous a-t-il déjà prescrit un traitement médicamenteux spécifique pour vous aider à réduire votre fatigue ? Et si oui, lequel ? MIROIR**.** *QCM sauf code 5 exclusif.* 1. Corticoïdes ; 2. Psychostimulant ; 3. Un autre traitement médicamenteux ; 4. Non, aucun traitement médicamenteux ; 5. Je ne sais pas.

Q25. Et vous-même avez-vous pris un traitement spécifique non prescrit par votre médecin pour soulager votre fatigue ? *QCM sauf code « Non » exclusive.* 1. Homéopathie ; 2. Vitamines
3. Autre : (*question ouverte) ;* 4. Non, je n'ai pris aucun traitement spécifique par moi-même.

Q26. Pour accompagner le traitement de votre fatigue, avez-vous bénéficié ou bénéficiez-vous de soins de support non médicamenteux ? Et si oui, lesquels ? MIROIR. *QCM – rotation aléatoire sauf code 10 + code 10 exclusif.* 1. Hypno-analgésie ; 2. Activité physique adaptée (APA) ; 3. Exercice physique ; 4. Acupuncture ; 5. Massage, relaxation ; 6. Consultation nutritionnelle ; 7. Accompagnement psychologique (thérapie cognitivo-comportementale, thérapie comportementale, Education Thérapeutique, Méditation Pleine Conscience) ; 8. Intervention sur les troubles du sommeil ; 9. Luminothérapie ; 10. Autre ; 11. Non, je n'ai bénéficié d'aucun des soins de support listés ci-dessus.

A ceux ayant répondu « Activité physique adaptée (APA) » : **Q27. Dans quelle(s) structure(s) pratiquez-vous ou avez-vous pratiqué cette activité physique adaptée (APA) ?** MIROIR**.** *QCM – rotation aléatoire sauf code 8.* 1. Au sein d'une association sportive ou dans une maison Sport Santé ; 2. A la maison avec un coach ; 3. Chez un kinésithérapeute ; 4. Dans un service de mon établissement ; 5. Dans un service en dehors de mon établissement ; 6. En autonomie (via des tutos, des applications) ; 7. Autre (*question ouverte*).

A ceux pratiquant au moins 1 soin de support : **Q28. A quel(s) moment(s) vous a-t-on proposé ces soins ?** MIROIR. *QCU.* 1. Au moment de l'annonce du cancer ; 2. Au cours du traitement du cancer.

Q29. Et pour accompagner le traitement de votre fatigue, vous a-t-on proposé d'avoir recours à une ou plusieurs pratiques complémentaires alternatives ? Et si oui, lesquelles ? *QCM – rotation aléatoire sauf code 11 et 12 + code 12 exclusif.* 1. Art-thérapie ; 2. Ostéo-thérapie ; 3. Sophrologie ; 4. Homéo-thérapie ; 5. Musicothérapie ; 6. Coupeur de feu ; 7. Aromathérapie ; 8. Réflexothérapie ; 9. Socio esthéticienne ; 10. Socio coiffeuse ; 11. Autre (*question ouverte*) ; 12. On ne m'a proposé aucune des pratiques complémentaires alternatives ci-dessus.

A ceux qui déclarent qu'on leur a proposé au moins une pratique complémentaire alternative + reprendre les items cochés : **Q30. De quelle(s) pratique(s) avez-vous bénéficié ?** *QCM – rotation aléatoire sauf code 11 et 12 + code 12 exclusif.* 1. Art-thérapie ; 2. Ostéo-thérapie ; 3. Sophrologie ; 4. Homéo-thérapie ; 5. Musicothérapie ; 6. Coupeur de feu ; 7. Aromathérapie ; 8. Réflexothérapie ; 9. Socio esthéticienne ; 10. Socio coiffeuse ; 11. Autre (*question ouverte*) ; 12. On ne m'a proposé aucune des pratiques complémentaires alternatives ci-dessus.

A ceux qui déclarent qu'on leur a proposé au moins une pratique complémentaire alternative : **Q31. Qui vous a proposé ce type de pratiques complémentaires alternatives ?** *QCM.* 1. Mon oncologue ; 2. Mon médecin traitant ; 3. Une association ; 4. L'infirmière d'hôpital de jour ou d'hospitalisation à domicile ; 5. Un proche ; 6. Un autre professionnel de santé.

A ceux qui déclarent qu'on leur a proposé au moins une pratique complémentaire alternative

Q32. A quel(s) moment(s) vous a-t-on proposé ces soins ? *QCU.* 1. Au moment de l'annonce du cancer ; 2. Au cours du traitement du cancer.

Q33. Au final, êtes-vous satisfait de la prise en charge de votre fatigue en lien avec votre cancer du sein métastatique ? MIROIR**.** *QCU.* 1. Oui, tout à fait ; 2. Oui, plutôt ; 3. Non, plutôt pas ; 4. Non, pas du tout.

Q34. Pouvez-vous préciser pourquoi ? *QO*

DIFFICULTES RENCONTREES PAR LES ONCOLOGUES
Q35. Selon-vous quelles sont les difficultés rencontrées par les professionnels de santé pour vous aider à gérer votre fatigue ? MIROIR. *QO.*

Q36. Et quelles seraient vos principales attentes concernant la prise en charge de votre fatigue en lien avec votre cancer du sein métastatique ? *QO*

PROFIL DES PATIENTS
Q37. Etes-vous ? *QCU.* 1. Un homme ; 2. Une femme.

Q38. Quel est votre âge ? *QNUM – borne 18-99 ans.* ***Recodage automatique de l'âge :*** 1. 18-24 ans ; 2. 25-34 ans ; 3. 35-44 ans ; 4. 45-54 ans ; 5. 55-64 ans ; 6. 65-74 ans ; 7. 75 ans et plus.

Q39. Dans quelle région vivez-vous ? *QCU.* 1. Grand Est ; 2. Nouvelle Aquitaine ; 3. Auvergne – Rhône-Alpes ; 4. Normandie ; 5. Bourgogne - Franche Comté ; 6. Bretagne ; 7. Centre-Val de Loire ; 8. Île-de-France ; 9. Occitanie ; 10. Hauts de France ; 11. Pays de la Loire ; 12. Provence-Alpes-Côte d'Azur, Corse.

Q40. Quelle est votre situation professionnelle actuelle ? *QCU.* 1. Agriculteur exploitant (à votre compte) ; 2. Artisan petit commerçant ; 3. Chef d'entreprise ; 4. Profession libérale (sauf paramédical) ; 5. Professeur / profession scientifique ; 6. Cadre et autre profession intellectuelle supérieure ; 7. Contremaître, agent de maîtrise, profession paramédicale, technicien ; 8. Instituteur ou assimilé ; 9. Employé ; 10. Personnel de service ; 11. Ouvrier / Ouvrier agricole ; 12. Retraité ; 13. Elève / étudiant ; 14. En arrêt de travail ; 15. Autre inactif (au foyer, dans une autre situation, …). *Recode automatique de la CSP en 3 tranches : 1. CSP+ (codes 1 à 8) ; 2. CSP- (codes 9 à 11) ; 3. Inactif (code 12 à 15) ; Recode automatique : 1. En activité (CSP+ et CSP-) ; 2. Inactif (code 3).*

Q41. Combien de personnes y compris vous comprend votre foyer ? *QNUM.* _ Personnes donc _ enfants.

Q42. Pouvez-vous indiquer ? *QNUM.* Votre poids en kg : _ _ _ kg ; Votre taille en cm : _ _ _ cm. *Recodage : calcul automatique de l'IMC.*

Q43. Est-ce que vous fumez ? *QCU.* 1. Oui, des cigarettes ou d'autres formes de tabac (pipe, cigare, chicha…) ; 2. Non, mais j'ai déjà fumé dans le passé ; 3. Non, je ne fume pas et je n'ai jamais fumé.

Q44. Consommez-vous de l'alcool ? *QCU.* 1. Oui, plusieurs fois par semaine ; 2. Oui, une fois par semaine ; 3. Oui, moins d'une fois par semaine ; 4. Oui, occasionnellement (évènements, noël…) ; 5. Non, jamais.

Q45. Pratiquez-vous une activité physique ou sportive aujourd'hui ? *QCU.* 1. Plus de 3 fois par semaine ; 2. 2 à 3 fois par semaine ; 3. Une fois par semaine ; 4. Moins souvent ; 5. Jamais.

À ceux qui pratiquent une activité physique ou sportive : **Q46. Pouvez-vous préciser quelle activité physique ou sportive pratiquez-vous aujourd’hui, le plus souvent ?** *QO.*

Q47. Pratiquiez-vous déjà cette activité avant votre diagnostic ? *QCU.* 1. Oui ; 2. Non.

Supplementary appendix 2 - Survey questionnaire. The dedicated questionnaires employed in the quantitative mirror study, conducted in France between July and September 2023 were answered by 143 metastatic breast cancer patients and 43 oncologists specializing in their treatment. These questionnaires featured a series of open or closed questions designed to capture data pertaining to the perspectives of both patients and physicians on fatigue and its treatment in metastatic breast cancer. Questions subjected to mirror-image analysis are identified by grey-colored lines.

**PATIENT QUESTIONAIRE**			**ONCOLOGIST QUESTIONNAIRE**		
STATUS OF THE DISEASE	Q1.	Are you or have you ever been personally affected by metastatic breast cancer?	SCREENER	Q1.	Are you an oncologist?
	Q2.	Is your current cancer a recurrence of a localized cancer, or is it metastatic from the start?		Q2.	Do you manage patients with metastatic breast cancer?
	Q3.	How long ago were you diagnosed with metastatic breast cancer?			
	Q4.	What treatments have you undergone to treat your metastatic breast cancer?			
	Q5.	Have you received trastuzumab deruxtecan as part of your treatment(s) for metastatic breast cancer?			
	Q6.	Where did your treatment sessions take place?			
EXPERIENCE AND IDENTIFICATION OF FATIGUE	Q7.	Can you indicate, on a scale of 0 to 10, how are you experiencing your care journey as part of the management of your metastatic breast cancer?	EXPERIENCE AND IDENTIFICATION OF FATIGUE	Q3.	On a scale of 0 to 10, what do you think is the experience of your patients with metastatic breast cancer?
	Q8.	Do you distinguish between physical and psychological fatigue?		Q4.	Do you distinguish among your patients with metastatic breast cancer what is physical fatigue and what is psychological fatigue?
	Q9.	For each stage of your treatment, what was your level of fatigue?		Q5.	For each stage of the treatment process, what is the level of fatigue in your patients with metastatic breast cancer?
	Q10.	When is fatigue most difficult to deal with?		Q6.	At what time(s) is physical fatigue and/or psychological fatigue most difficult for your patients with metastatic breast cancer?
	Q11.	Here's a list of characteristics that can explain fatigue, for most people (*Cancer treatments, Anxiety, My sleep problems, The pain, Feeling diminished and vulnerable, Organizational constraints, Reducing my physical activity, My body's defense reactions, Changes in my weight or muscle mass, Disease progression, Depression, The increase in my sedentary lifestyle, Anemia, Difficulties eating, Another reason*). Can you rank in descending order the 5 main causes of fatigue that you feel or have felt?		Q7.	Here's a list of characteristics that can explain fatigue, for most people (*Cancer treatments, Physical deconditioning, Anxiety, Sleep disorders, Pain, Depression, Organizational constraints, Reduced physical activity, undernutrition, Changes in weight and body muscle mass, Tumor disease progression, Associated pathologies and comorbidities, Increased sedentary style, Anemia, Inflammatory cytokine production, Cognitive, behavioral and motivational factors, Psycho-social context*). Can you rank in descending order the 5 main causes of fatigue experienced by your metastatic breast cancer patients?
	Q12.	Have you ever spoken to a healthcare professional about your fatigue related to your metastatic breast cancer?		Q8.	Would you say that your patients with metastatic breast cancer talk to you or ask you to help them manage their fatigue?
	Q13.	With which healthcare professional(s) have you discussed your fatigue related to your metastatic breast cancer?			
	Q14.	Why have you never spoken to a healthcare professional about your fatigue related to your metastatic breast cancer?			
	Q15.	Here are several elements related to well-being (*Side effects of treatment, lack of energy, Impact on professional and domestic activities, Impaired sleep, realization of the deterioration in condition, Pain, impact on intimate life, Sadness, Loss of hope in the fight against the disease, Strong feeling of being sick, Nausea, Inability to assume one's role in the family, Nervousness, Absence or lack of friendly and family support, Isolation, Denial of illness by family, Rejection of work, Distance from friends, Disinterest in leisure, Distance from partner, Denial of illness by the patient, Dissatisfaction with how the illness is managed, Bed rest*) that may have been impacted by fatigue related to your cancer. Can you rank the 5 items that impact you the most?		Q9.	Here are several elements related to well-being (*Side effects of treatment, lack of energy, Impact on professional and domestic activities, Impaired sleep, realization of the deterioration in condition, Pain, impact on intimate life, Sadness, Loss of hope in the fight against the disease, Strong feeling of being sick, Nausea, Inability to assume one's role in the family, Nervousness, Absence or lack of friendly and family support, Isolation, Denial of illness by family, Rejection of work, Distance from friends, Disinterest in leisure, Distance from partner, Denial of illness by the patient, Dissatisfaction with how the illness is managed, Bed rest*) that can be impacted by cancer-related fatigue. Can you classify the 5 major items which, according to you, are impacted by fatigue in your patients?
MANAGING FATIGUE	Q16.	On a scale of 0 to 10, how effective would you rate the management of your fatigue linked to your metastatic breast cancer?	MANAGING FATIGUE	Q10.	In your opinion, on a scale of 0 to 10, how would you rate the effectiveness of the management of fatigue linked to breast cancer in your patients at the metastatic stage?
	Q17.	Has your doctor offered you an assessment of your fatigue related to your metastatic breast cancer?		Q11.	Do you offer a fatigue assessment to your patients with metastatic breast cancer?<br>How would you define this role?
	Q18.	How did your doctor assess your fatigue related to your metastatic breast cancer?		Q12.	How do you assess the fatigue level of your patients with metastatic breast cancer?.
	Q19.	What does the oncologist recommend to help you relieve the fatigue linked to your metastatic breast cancer on your own?		Q13.	What biological tests do you perform to consider the different components of fatigue?
				Q14.	What clinical assessment(s) do you perform to consider the different components of fatigue?
				Q15.	Do you take treatment history (metastatic breast cancer or not) into account to anticipate the occurrence of fatigue in your patients with metastatic breast cancer?
				Q16.	Which of the following tools or aids* are you familiar with, even if only by name, to measure the fatigue level of your patients with metastatic breast cancer? **QLQ-C30, VAS digital scale, Brief Fatigue Inventory, FACIT, Fatigue severity scale, INCa questionnaire, QLQ-FA12, Daily Fatigue Cancer Scale, Multidimensional Fatigue Inventory VAS digital scale, Modified Piper, Multidimensional Fatigue Inventory, Other*
				Q17.	And among the tools you know, which ones do you use to measure the level of fatigue in your patients with metastatic breast cancer?
				Q18.	Why do you not use tools or supports to assess the level of fatigue in your patients with metastatic breast cancer?
	Q20.	Would you say that your oncologist has a role to play in managing your fatigue related to your metastatic breast cancer?		Q19.	Would you say that you have a role to play in the management of fatigue in your patients with metastatic breast cancer?
				Q20.	How would you define this role?
	Q21.	Did other professionals play a role in managing your fatigue?			
	Q22.	And would you say that your oncologist takes your opinion and your feelings regarding your fatigue sufficiently into account?			
	Q23.	Has your oncologist ever modified or adjusted your cancer treatment to reduce your level of fatigue?		Q21.	Do you ever modify or adjust the anticancer treatments of your patients with metastatic cancer to reduce fatigue?
	Q24.	Has your oncologist ever prescribed any specific medication to help reduce your fatigue? And if so, which one?		Q22.	Have you ever prescribed a specific medication treatment to help reduce fatigue and associated symptoms in your patients with metastatic breast cancer? And if so, which one(s)?
	Q25.	And have you, yourself, taken any specific treatment not prescribed by your doctor to relieve your fatigue?			
	Q26.	To support the treatment of your fatigue, have you benefited or are you benefiting from non-drug supportive care? And if so, which ones (*Adapted physical activity, Physical exercise, Massage, relaxation, Acupuncture, Psychological support, Nutritional consultation, Intervention for sleep disorders, Light therapy, Hypno-analgesia, Other*)?		Q23.	To support the treatment of fatigue, do you offer your metastatic breast cancer patients non-drug supportive care? And if so, which ones (*Adapted physical activity, Physical exercise, Massage, relaxation, Acupuncture, Psychological support, Nutritional consultation, Intervention for sleep disorders, Light therapy, Hypno-analgesia, Other*) ?
	Q27.	In which structure(s) do you practice or have you practiced this adapted physical activity (APA)?		Q24.	To which structure(s) do you refer your patients for adapted physical activity (APA)?.
	Q28.	When were you offered this treatment?		Q25.	When do you most often offer this type of care to your patients with metastatic breast cancer?
	Q29.	And to support the treatment of your fatigue, were you offered one or more complementary alternative practices? And if so, which ones?			
	Q30.	What practice(s) have you benefited from?			
	Q31.	Who suggested this type of alternative complementary practice to you?			
	Q32.	When were you offered these treatments?			
	Q33.	Ultimately, are you satisfied with the management of your fatigue related to your metastatic breast cancer?		Q26.	Ultimately, are you satisfied with the management of fatigue in your patients with metastatic breast cancer?
	Q34.	Can you clarify why?			
DIFFICULTIES ENCOUNTERED BY ONCOLOGISTS	Q35.	In your opinion, what are the difficulties encountered by health professionals in helping you manage your fatigue?	DIFFICULTIES ENCOUNTERED BY ONCOLOGISTS	Q27.	What difficulties do you encounter in the management of fatigue in your patients with metastatic breast cancer?
	Q36.	And what would be your main expectations concerning the management of your fatigue related to your metastatic breast cancer?		Q28.	In your opinion, what do you think should be done as a priority to improve the management of fatigue in your patients with metastatic breast cancer?
PATIENT PROFILE	Q37.	Are you a patient?	PROFILE OF ONCOLOGISTS	Q29.	What is your gender?
	Q38.	How old are you?		Q30.	What is your age?
	Q39.	Where do you live?		Q31.	Do you have a hospital-based or mixed practice?
	Q40.	What is your current employment status?		Q32.	What percentage of your work is hospital-based?
	Q41.	How many people are in your household, including yourself?		Q33.	What type of establishment do you work in?
	Q42.	Can you tell us?		Q34.	In which region do you work?
	Q43.	Do you smoke?			
	Q44.	Do you drink alcohol?			
	Q45.	Do you practise any physical activity or sport today?			
	Q46.	If yes: Can you specify what physical or sporting activity you currently practise most often?			
	Q47.	If yes: Did you already practise this activity before your diagnosis?			

Supplementary Appendix 3: The French National Cancer Institute's (INCa) questionnaire (38).

Example questionnaire to record symptoms of fatigue

The following scale is suggested:

0= no symptoms
1= very infrequent
2= quite frequent
3= frequent

	**Dates** (specify the time of day: morning, midday, evening etc.)					
	**Symptoms**					
Do you get out of breath?						
Do you get tired quickly?						
At what time of the day are you most tired?						
Do you have trouble getting to sleep?						
Do you wake-up in the night? What wakes you (anxiety, pain etc.)?						
Have you lost your appetite?						
Do you have pains in your arms, legs, shoulders?						
Do you feel that your heart beats too fast?						
Do you have digestive problems (diarrhea, constipation etc.)?						
Do you have trouble concentrating?						
Do you feel anxious, depressed?						
Do you find it difficult to carry out daily tasks either at home or work?						
Other						

Depending on the different symptoms described (excessive fatigue, pain, poor quality sleep, feelings of depression) and the effect they have on the patient's quality of life, pharmacological treatment may be considered.

Supplementary appendix 4 – TableS1: Impact of fatigue on patients' daily lives.
Responses to; Patient's question: "*Here are several elements related to well-being that may have been impacted by fatigue related to your cancer. Can you rank the 5 items that impact you the most?*" Doctor's question: "*Here are several elements related to well-being that can be impacted by cancer-related fatigue. Can you classify the 5 major items which, according to you, are impacted by fatigue in your patients?*". There is considerable divergence in some domains, with doctors underestimating the impact of fatigue on family and functional well-being and concentrating on the physical part of the disease. The table below gives the details within each domain.

**Physical well-being**		**Emotional well-being**		**Functional well-being**		**Family well-being**	
Side effects of treatment	Drs Pts 74% 61%	Realization that health is deteriorating	Drs Pts 49% 46%	Impact on professional & domestic activities	Drs Pts 37% 50%	Impact on intimate life	Drs Pts 7% 33%
Lack of energy	Drs Pts 40% 59%	Sadness	Drs Pts 23% 28%	Impaired sleep	Drs Pts 44% 49%	Absence or lack of friendly & family	Drs Pts 44% 49%
Pain	Drs Pts 56% 38%	Loss of hope in battle against the disease	Drs Pts 40% 24%	Rejection of work	Drs Pts 5% 7%	Denial of illness by family	Drs Pts 5% 10%
Enhanced feeling of being ill	Drs Pts 42% 17%	Nervousness	Drs Pts 5% 12%	Disinterest in leisure	Drs Pts 7% 5%	Isolation	Drs Pts 9% 10%
Nausea	Drs Pts 42% 17%	Dissatisfaction with management of treatment	Drs Pts 2% 1%	Denial of illness by the patient	Drs Pts 0% 2%	Distance from friends	Drs Pts 5% 6%
Inability to fulfill role in the family	Drs Pts 12% 13%					Distance from friends	Drs Pts 9% 4%
Bed rest	Drs Pts 9% 0%						
